# Pericytes as key mediators of microenvironmental signaling crosstalk: implications in vascular diseases and cancer

**DOI:** 10.3389/fcell.2026.1899684

**Published:** 2026-08-13

**Authors:** Qi Guo, Yujia Yao, Yueying Ding, Lushu Chen, Qiuyang Zhang, Huiying Zhang, Jinsong Xue

**Affiliations:** 1 Affiliated Eye Hospital, Nanjing Medical University, Nanjing, China; 2 The Fourth School of Clinical Medicine, Nanjing Medical University, Nanjing, China

**Keywords:** pericytes, signaling integration, tumor microenvironment, vascular barrier, vascular remodeling

## Abstract

Pericytes are important components of microenvironmental signaling interactions within the microvascular wall and participate in maintaining vascular stability and regulating endothelial function. They are increasingly recognized as important components of the vascular microenvironment rather than isolated structural cells. Under physiological conditions, they integrate biochemical, mechanical, and immune signals to preserve vascular barrier integrity and support vessel remodeling, functioning as one component of a broader multicellular network that includes endothelial cells, astrocytes, smooth muscle cells, immune cells, and extracellular matrix components. Pathological stimuli including metabolic stress, ischemia, or oncogenic signaling disrupt these regulatory networks. As a result, pericy-mediated interactions undergo adaptive remodeling, contributing to diverse vascular phenotypes. These range from hyperpermeability in retinal and neurovascular disorders to structurally and functionally abnormal tumor vasculature. In this review, we explore the mechanisms by which pericytes participate in signaling crosstalk in health and disease, emphasizing their interactions with endothelial cells, immune cells, and extracellular matrix components. Additionally, we provide an overview of the potential therapeutic implications of targeting pericyte-mediated signaling to restore crosstalk balance and achieve vascular normalization. These approaches position pericytes as important signaling nodes within microenvironmental signaling networks and provide a conceptual foundation for more effective interventions in vascular diseases and cancer.

## 1 Introduction

The microvasculature plays an important role in maintaining tissue homeostasis by regulating the exchange of nutrients, metabolites, and signaling molecules between the circulation and surrounding tissues (Antonetti, 2009). Specialized vascular barriers, such as the blood-brain barrier (BBB) and blood-retinal barrier (BRB), are essential for preserving tissue-specific microenvironments and protecting against pathological insults (Armulik et al., 2011). Disruption of these barriers is a hallmark of numerous diseases, including diabetic retinopathy, stroke, neurodegeneration, and cancer (Antonetti et al., 2006; Rao and Dlouhy, 2013; Sweeney et al., 2019; Zlokovic, 2011).

Pericytes, as mural cells of the microvasculature, are increasingly recognized as important participants in vascular homeostasis and disease. They contribute to the regulation of vascular tone, endothelial junctional stability, angiogenesis, immune regulation, and metabolic coupling (Vanlandewijck et al., 2018b). Their strategic anatomical positioning and functional plasticity enable them to integrate biochemical, mechanical, and inflammatory signals, thereby dynamically modulating barrier properties (Bell et al., 2023; Zlokovic, 2011). However, pericytes exhibit considerable heterogeneity across vascular beds and organs, and their precise roles in barrier maintenance and disease progression are still being unraveled (Attwell et al., 2016; Vanlandewijck et al., 2018b). This heterogeneity is influenced by the diverse microenvironmental cues that pericytes encounter, including metabolic signals, inflammatory mediators, mechanical forces, and extracellular matrix-derived inputs, all of which vary significantly across physiological and pathological contexts. While individual signaling pathways such as PDGF, vascular endothelial growth factor (VEGF), and Ang/Tie2 have been extensively studied, how these diverse inputs are integrated to shape vascular responses remains poorly understood. This gap has limited our ability to fully explain the heterogeneity of vascular dysfunction observed across different diseases (Obermeier et al., 2013; Profaci et al., 2020).

Importantly, pericytes do not function in isolation. The vascular microenvironment comprises a complex network of interacting cell types including endothelial cells, astrocytes, vascular smooth muscle cells, pericytes, immune cells, and fibroblasts, as well as the extracellular matrix (Garcia-Martínez et al., 2025). Many of the signaling pathways discussed in this review are bidirectional and involve multiple cell types; pericytes represent one important component of this broader multicellular network rather than the sole or dominant regulator. Throughout this review, we have sought to reflect this complexity by discussing pericyte functions in the context of their multicellular interactions (Wang et al., 2025).

While vascular diseases and cancer are often considered distinct pathological entities, they share fundamental mechanisms of microvascular dysfunction that are mediated, at least in part, by pericyte dysregulation. In retinal and neurovascular disorders, pericyte loss or dysfunction is associated with barrier hyperpermeability, inflammatory cell infiltration, and tissue damage (Yu et al., 2025). In cancer, pericytes exhibit abnormal coverage, altered signaling, and defective interactions with endothelial cells, contributing to vascular leakiness, hypoxia, and impaired drug delivery. Despite these seemingly opposite manifestations, namely, barrier dysfunction *versus* abnormal vessel formation, both contexts involve disrupted pericyte-mediated signaling crosstalk with endothelial cells, immune cells, and the extracellular matrix (He et al., 2025). Convergent signaling pathways, including PDGF-B/PDGFRβ, Ang/Tie2, TGF-β, and VEGF/VEGFR axes, are implicated across both vascular barrier diseases and tumor vascular biology. This shared mechanistic foundation supports examining pericyte biology across vascular barrier disorders and tumor-associated vascular remodeling within a single framework (Yamazaki et al., 2016). By systematically comparing how pericyte signaling networks are rewired across these conditions, we aim to identify convergent mechanisms and therapeutic principles that may inform treatment strategies for a broad spectrum of diseases.

This review aims to synthesize recent advances in pericyte biology, with a focus on their roles as important signaling nodes in microenvironmental signaling crosstalk across vascular, neurovascular, and tumor-associated diseases. Through interactions with endothelial cells, immune cells, and the extracellular matrix, pericytes participate in the regulation of vascular barrier states across a spectrum from homeostasis to dysfunction. Importantly, this crosstalk is highly context-dependent, often associated with distinct vascular phenotypes under metabolic, neurovascular, and tumor-associated conditions (Armulik et al., 2011; Sweeney et al., 2016; Vanlandewijck et al., 2018b; Zlokovic, 2011). We also outline the mechanistic basis of pericyte-mediated crosstalk, encompassing structural interactions, paracrine signaling, immune modulation, mechanotransduction, and extracellular vesicle-mediated communication. We then examine how these mechanisms are differentially rewired across disease contexts, with a focus on retinal and neurovascular disorders. Finally, we highlight the tumor microenvironment as an extreme model of crosstalk reprogramming, in which multiple signaling axes converge to contribute to vascular dysfunction, immune evasion, and therapeutic resistance. By framing pericytes as important signaling nodes within multicellular networks rather than isolated regulators, this review provides a cohesive perspective on vascular biology and identifies new opportunities for therapeutic intervention across a broad spectrum of diseases.

## 2 Identity and heterogeneity of pericytes

### 2.1 Pericyte definition and basic characteristics

**FIGURE 1:**
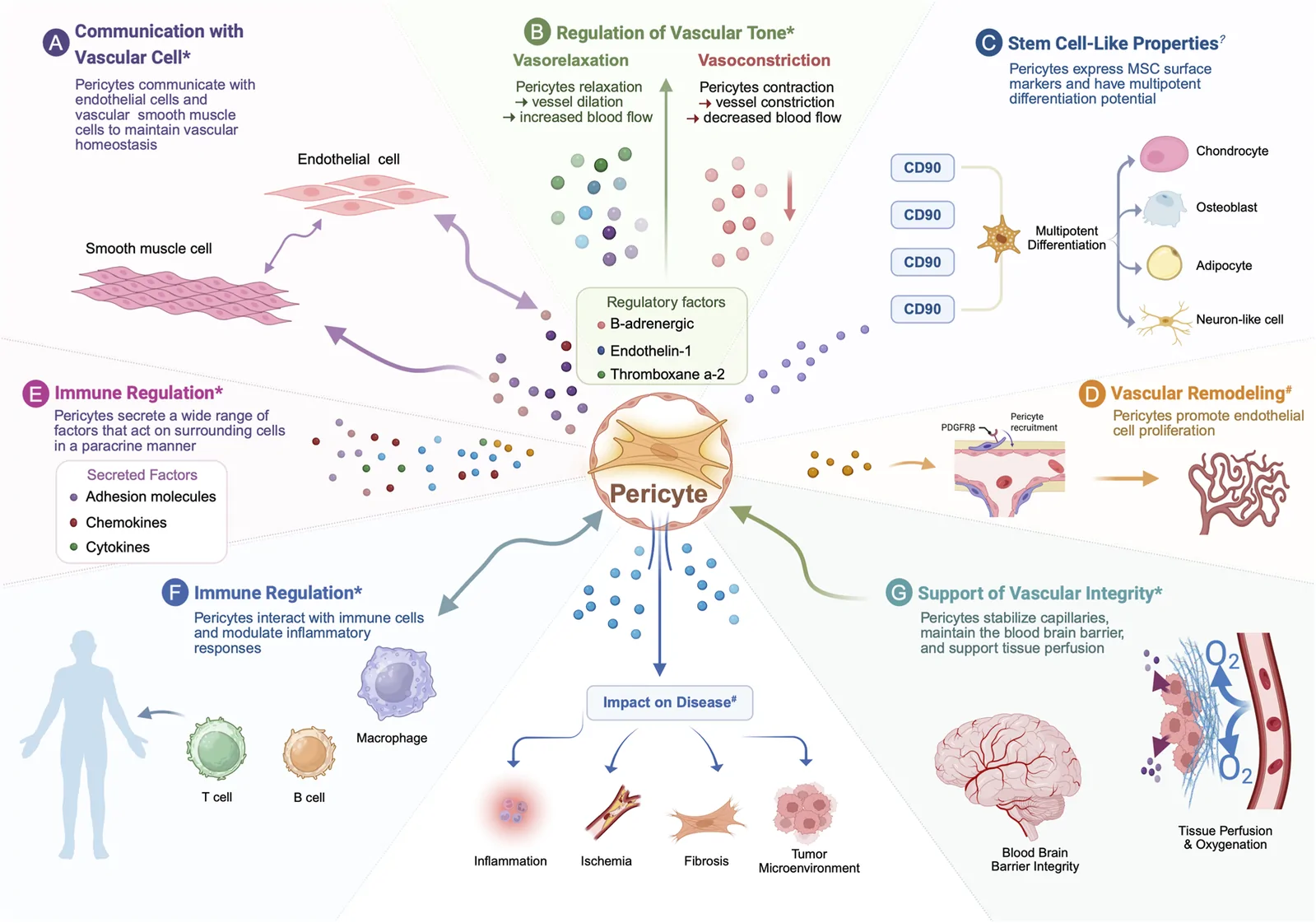
Pericytes maintain vascular homeostasis and modulate inflammatory responses through bidirectional communication with endothelial cells, smooth muscle cells, and immune cells. Interactions depicted in this figure are annotated according to the level of evidence: strong experimental evidence is indicated by an asterisk (*), well-supported mechanistic frameworks are marked with a hash symbol (#), and emerging or hypothetical concepts are indicated by a question mark (?).

Pericytes are mural cells embedded in the microvascular basement membrane and positioned abluminal to endothelial cells, where they are widely distributed along capillaries and postcapillary venules (Bodnar et al., 2013; Wu et al., 2023) (Figure 1). Pericytes originate from multiple mesenchymal sources. In most tissues, they derive from the mesoderm, whereas in the head and central nervous system, a significant proportion pericytes arises from the neural crest. Additionally, evidence suggests that some pericytes can differentiate from mesenchymal stem cells and retain the capacity for multilineage differentiation under specific conditions.

Accurate identification of pericytes remains challenging and continues to be actively debate in the field, largely owing to the lack of highly specific and universally accepted molecular markers (Gonzales et al., 2020). Commonly used pericyte markers include platelet-derived growth factor receptor-β (PDGFRβ), neuron glial antigen 2 (NG2, CSPG4), CD146, and Desmin (Bohannon et al., 2025). However, the expression patterns of these markers substantially overlaps between pericytes, vascular smooth muscle cells (vSMCs) and fibroblasts, rendering single-marker strategies insufficient for reliable pericyte definition. For instance, PDGFRβ expression exists along a continuum rather than defining a discrete boundary between these populations (Roedel et al., 2025; Wu et al., 2023; Zhao and Lederer, 2025). Similarly, α-smooth muscle actin (α-SMA), frequently used as a vSMC marker, is also expressed by certain pericyte subpopulations, particularly those at the arteriolar-capillary transition, under both physiological and pathological conditions. Furthermore, the expression of markers such as NG2 and CD146 exhibits substantial variability depending on tissue type, developmental stage, and disease context (Zhao and Lederer, 2025). These observations indicate that pericytes comprise multiple subpopulations with distinct structural and functional properties and display tissue-specific distribution patterns: they are highly abundant in the brain and retina, where they are essential for maintaining BBB and BRB integrity; they contribute to blood flow regulation and vascular remodeling in the heart, lung, kidney, and skeletal muscle; and in tumors, they are often present but show abnormal morphology, reduced coverage, and altered signaling, contributing to dysfunctional tumor microvessels and impaired drug delivery (Lin et al., 2025). Collectively, the lack of highly specific, universally accepted markers remains a major controversy in pericyte biology and complicates the interpretation of studies relying on single-marker strategies. Consensus recommendations have proposed multi-marker combinatorial approaches, combined with functional and anatomical criteria, to improve the rigor of pericyte identification; however, implementation across the field remains inconsistent. This structural and functional heterogeneity enables pericytes to adapt to the specific demands of diverse vascular and tissue environments.

### 2.2 Morphological and functional heterogeneity of pericytes

Pericytes exhibit substantial morphological diversity along the vascular tree. Based on their process architecture and coverage pattern, they are broadly categorized into three subtypes: ensheathing, mesh, and thin-strand pericytes (Abdelazim et al., 2022). Ensheathing pericytes, typically found at arteriolar branch points and precapillary sphincters, wrap tightly around the vessel wall and exhibit high contractile capacity. They are therefore proposed to contribute to local blood flow regulation, although the extent of their contractile function remains context-dependent. Mesh pericytes, characterized by a net-like arrangement of processes, are more prevalent along mid-capillary segments and are thought to provide structural support and regulate junctional stability. Thin-strand pericytes, located on distal capillaries, possess elongated, minimally branched processes and are more involved in paracrine signaling and immune surveillance than in mechanical regulation (Ippolitov et al., 2022). These morphological distinctions are not static; under pathological stress, pericytes can undergo shape changes and process retraction, which are associated with barrier opening and vascular leakage (Li and Fan, 2023).

In addition to morphological heterogeneity, pericytes display tissue-specific functional specialization. In the brain and retina, pericytes contribute to BBB and BRB maintenance, where they regulate tight junction expression *via* Hedgehog and TGF-β signaling (Sun et al., 2021). In the heart, lung, kidney, and skeletal muscle, pericytes contribute more prominently to vascular tone, compliance, and post-injury remodeling, with a greater emphasis on mechanotransduction and extracellular matrix (ECM) production (Lee and Chintalgattu, 2019). In tumors, pericytes are often present but display aberrant phenotypes, including reduced coverage, disorganized basement membrane deposition, and altered secretion of angiogenic factors, which collectively promote vessel tortuosity, hyperpermeability, and impaired perfusion (Stout, 1956). These tissue-specific differences underscore the importance of interpreting pericyte function within the appropriate organ context.

Emerging single-cell transcriptomic and lineage-tracing studies have further revealed disease-associated pericyte states that are not observed under homeostasis. In conditions of metabolic stress, ischemia, or chronic inflammation, pericytes upregulate inflammatory gene programs, fibrotic markers, and angiogenic factors (Gao et al., 2026). In some contexts, pericytes may transition into myofibroblasts and contribute to tissue fibrosis, while in others they acquire a senescent-like phenotype, further exacerbating barrier dysfunction (Li et al., 2024a). Importantly, these states are not fixed; evidence suggests bidirectional plasticity between inflammatory and fibrotic programs, as well as interconversion between different pericyte subtypes, indicating that pericyte heterogeneity is a dynamic and context-dependent continuum rather than a rigid classification (Schuldt et al., 2025).

A key challenge in studying pericyte heterogeneity lies in the limitations of current marker-based definitions. Widely used markers such as PDGFRβ, NG2, CD146, and Desmin are also expressed on vascular smooth muscle cells, fibroblasts, and even some immune cells, leading to significant overlap and potential misidentification (Li et al., 2024b). Single-marker strategies are insufficient, and even combinatorial approaches require careful validation across tissues and disease states. Recent efforts have employed single-cell RNA-seq to define pericyte-specific transcriptomic signatures, but consensus is still lacking. Therefore, a functional and contextual framework for defining pericytes, combining anatomical location, morphological criteria, and a panel of molecular markers, while acknowledging that no single definition is universally applicable. This integrated approach is essential for correctly interpreting the diverse roles of pericytes in vascular homeostasis and disease.

## 3 Conceptual framework of pericyte-mediated signaling crosstalk

Pericytes function as important regulatory nodes that integrate biochemical, mechanical, and inflammatory cues within a multilayered microenvironmental signaling network (Winkler et al., 2011; Winkler et al., 2017). They engage in dynamic crosstalk with endothelial cells to regulate junctional stability and permeability, while also communicating with immune cells and remodeling the extracellular matrix (ECM) in response to local cues (Procter et al., 2021). Through an integrated multilayered network encompassing endothelial, immune, and ECM interactions, pericytes contribute to the regulation of vascular barrier homeostasis and modulate barrier function across diverse physiological and pathological conditions (Table 1) (Armulik et al., 2005).

**TABLE 1:** Signaling pathways involved in pericyte-mediated vascular regulation and the corresponding evidence strength in disease contexts.

Signaling pathway	Key molecules	Pericyte function	Disease relevance	Evidence strength
PDGF-B/PDGFRβ	PDGF-B ligand; PDGFRβ receptor	Pericyte recruitment and survival; endothelial-pericyte attachment	Vascular development; BBB/BRB stability; tumor vessel abnormality	Established
VEGF/VEGFR axis	VEGF; VEGFR1 (pericyte decoy)	Permeability regulation; endothelial activation	Diabetic retinopathy; hypoxia; tumor leakiness	Established
Angiopoietin–Tie2	Ang-1/Ang-2; Tie2 receptor	Vessel stabilization vs destabilization	Inflammation; DR; vascular leakage	Established
TGF-β signaling	TGF-β; ALK5 receptor	Tight junction & ECM regulation	Barrier integrity; fibrosis	Established
Notch signaling	Jagged1–Notch3	Endothelial maturation; vessel stabilization	Angiogenesis; vascular remodeling	Established
Hedgehog signaling	Sonic hedgehog pathway	Tight junction induction	BBB/BRB maintenance	Established
PI3K/Akt pathway	Downstream of Ang-1/Tie2	Endothelial survival; anti-permeability	Inflammation; hypoxia	Supported working model
NF-κB / MAPK	TNF-α, IL-1β induced pathways	Inflammatory activation in pericytes	Barrier breakdown; immune recruitment	Supported working model
AGE–RAGE axis	AGEs binding RAGE → NADPH oxidase	Oxidative stress & inflammation	Diabetic retinopathy microangiopathy	Established
Polyol pathway	Aldose reductase; sorbitol accumulation	Redox imbalance; osmotic stress	Hyperglycemia-induced pericyte injury	Supported working model
PKC signaling	Protein kinase C activation	Permeability & ECM remodeling	DR vascular dysfunction	Established
HIF-1α pathway	Hypoxia-induced HIF-1α	VEGF transcription activation	Ischemia-driven angiogenesis	Established
RhoA/ROCK	Small GTPase signaling	Contractility & cytoskeletal tension	Mechanotransduction; leakage	Established
YAP/TAZ	Hippo mechanotransduction effector	ECM stiffness sensing	Fibrosis; vascular remodeling	Supported working model
Piezo1	Mechanosensitive ion channel	Shear stress sensing	Capillary flow regulation	Emerging
TRP channels	TRPC3; TRPM4/7	Mechanical stress ion signaling	Cerebral flow; ischemic injury	Supported working model
GPCR mechanosensing	ADGRG1 and adhesion GPCRs	Force-responsive signaling	Vascular remodeling	Emerging
MMPs / ECM remodeling	MMP-2/9; TIMPs	Basement membrane degradation	Barrier disruption; angiogenesis	Established
Extracellular vehicles (EVs)	miRNAs, proteins, lipids	Intercellular communication	DR; inflammation; vascular repair	Emerging

### 3.1 Physical support and structural maintenance

Pericytes are known to provide a fundamental structural framework that maintains vascular barrier stability by forming structurally coupled interfaces with endothelial cells and remodeling the basement membrane microenvironment (Bergers and Song, 2005; Daneman et al., 2010). Multiple forms of direct physical contacts between these 2 cell types include peg-and-socket junctions, focal adhesions, and gap junctions (Berthiaume et al., 2018; Hartmann et al., 2021). These structures are mediated by adhesion molecules comprising N-cadherin, integrins, and connexins, which reinforce intercellular adhesion and facilitating bidirectional mechanotransduction (Kisler et al., 2017; Nikolakopoulou et al., 2019; Török et al., 2021). Gap junctions formed by connexins not only support structural coupling but also function as conduits for ions, small signaling molecules (e.g., cAMP, IP), and electrical signals between pericytes and endothelial cells, thereby enabling rapid intercellular signal transmission.

These structural interactions are embedded within the basement membrane, whose composition and architecture are in turn co-regulated by pericytes and endothelial cells (Nzou et al., 2018). Pericytes represent a major source of key extracellular matrix components, including laminins, type IV collagen, and heparan sulfate proteoglycans (Mathias et al., 2025; Smyth et al., 2018). They participate in basement membrane synthesis, degradation, and dynamic remodeling by modulating the expression of matrix metalloproteinases and their inhibitors (Ribatti et al., 2021; Underly et al., 2017). These interactions contribute to mechanical support that helps maintain endothelial anchorage, while preserving flexibility and plasticity that accommodate physiological vascular remodeling and microenvironmental changes (Kemp et al., 2020). Through these structural interactions, pericytes regulate endothelial cytoskeletal tension, morphology, and junctional organization, effectively preventing abnormal expansion of paracellular spaces.

When pericyte coverage is insufficient or structural coupling with endothelial cells is disrupted, basement membrane continuity deteriorates, integrin-mediated adhesive signaling weakens (Bu et al., 2019). Tight and adherens junction complexes subsequently disassemble, ultimately leading to dilation of paracellular pathways and increased vascular permeability (Kang et al., 2019; Yang et al., 2022). These alterations are particularly evident in pathological conditions such as diabetic microangiopathy, tumor vascular abnormalities, and neurovascular unit dysfunction, thereby compromising microvascular structural integrity and impairing vascular barrier function (Banks et al., 2021; Chen et al., 2020).

The role of pericytes as contractile cells regulating capillary blood flow remains an area of ongoing debate. Early studies identified contractile proteins in pericytes, including α-SMA, tropomyosin, and myosin. *In vitro* experiments demonstrated contraction in response to vasoactive mediators such as endothelin-1, angiotensin II, and ATP (Wilhelm et al., 2025). *In vivo* imaging in the brain and retina further suggested that pericytes may contribute to capillary diameter regulation and local blood flow control (Erdener et al., 2022); however, the physiological relevance and magnitude of these effects remain contested. Alternative evidence proposes that capillary-level flow regulation is primarily governed by upstream arteriolar smooth muscle cells, with pericytes acting in a more modulatory or context-dependent manner (Hill et al., 2015). This controversy is further compounded by methodological differences across studies (e.g., *in vivo* two-photon imaging, intrinsic optical signal approaches, and *in vitro* vs. *in vivo* systems), as well as pericyte heterogeneity. In particular, ensheathing pericytes at arteriolar-capillary transitions appear more contractile than thin-strand pericytes in distal capillaries, and most supporting *in vivo* evidence derives from CNS studies, with peripheral vascular beds remaining less well characterized (Alarcon-Martinez et al., 2019). Collectively, pericyte contractility is best viewed as a context-dependent and subtype-specific property influenced by vascular location and experimental conditions.

### 3.2 Regulation of vascular homeostasis mediated by secreted factors

Pericytes not only provide structural support to microvessels but also serve as important signaling interactions, sustaining and reinforcing vascular barrier function through the continuous secretion of diverse paracrine factors (Hall et al., 2014; Hill et al., 2015). Under physiological conditions, pericytes secrete factors such as TGF-β, angiopoietin-1 (Ang-1), and Notch ligands (Dight et al., 2022; Hogan et al., 2009). These factors collectively promote endothelial cells to adopt a low-permeability phenotype with stable junctional architecture, thereby effectively restricting molecular diffusion (Potente and Mäkinen, 2017). Pericyte-derived TGF-β enhances the expression and proper assembly of endothelial tight junction proteins, including claudin-5, occludin, and ZO-1, while strengthening cell basement membrane adhesion (Dave et al., 2018; Walshe et al., 2011). Pericytes release Ang-1, which binds its receptor Tie2 on endothelial cells to activate the PI3K/Akt pathway. This signaling is associated with stabilization of endothelial junctional complexes and counteracting pro-permeability stimuli under inflammatory or hypoxic conditions, thus maintaining vascular low-permeability states (Joussen et al., 2021; Souma et al., 2018). Notch signaling, activated *via* ligands such as Jagged1, releases the intracellular domain NICD, which suppresses transcriptional programs associated with vascular activation and increased permeability (Cao et al., 2017). As a result, endothelial junctions mature and barrier homeostasis is maintained. Additionally, Notch signaling restricts aberrant vascular sprouting and accelerates the maturation of nascent vessels, thereby preserving overall vascular network stability (Pitulescu et al., 2017).

Pericytes also finely tune local microenvironmental cues that regulate vascular permeability, particularly through the modulation of VEGF (Eilken et al., 2017). By expressing high affinity but weakly signaling VEGFR1 on their surface, pericytes sequester VEGF and mitigate its stimulatory effects on endothelial cells (Langen et al., 2017). VEGF further binds heparan sulfate proteoglycans (HSPGs) within the extracellular matrix, stabilizing extracellular VEGF gradients and modulating its spatial distribution (Pérez-Gutiérrez and Ferrara, 2023). This mechanism establishes a relatively stable perivascular microenvironment that constrains increases in vascular permeability. These secreted factors mediate paracrine crosstalk between pericytes and endothelial cells. Loss of pericyte coverage or functional impairment is associated with excessive VEGF-driven activation of endothelial permeability pathways. This triggers degradation of tight junction proteins and VE-cadherin internalization, ultimately disrupting adherens junctions and elevating barrier permeability (Park et al., 2016). Collectively, these observations suggest that pericytes are involved in pro-survival signaling and barrier-stabilizing programs, ensuring the structural and functional integrity of the vascular network (Nikolakopoulou et al., 2019; Trempel et al., 2025).

### 3.3 Modulation by inflammatory responses and mechanical cues

**FIGURE 2:**
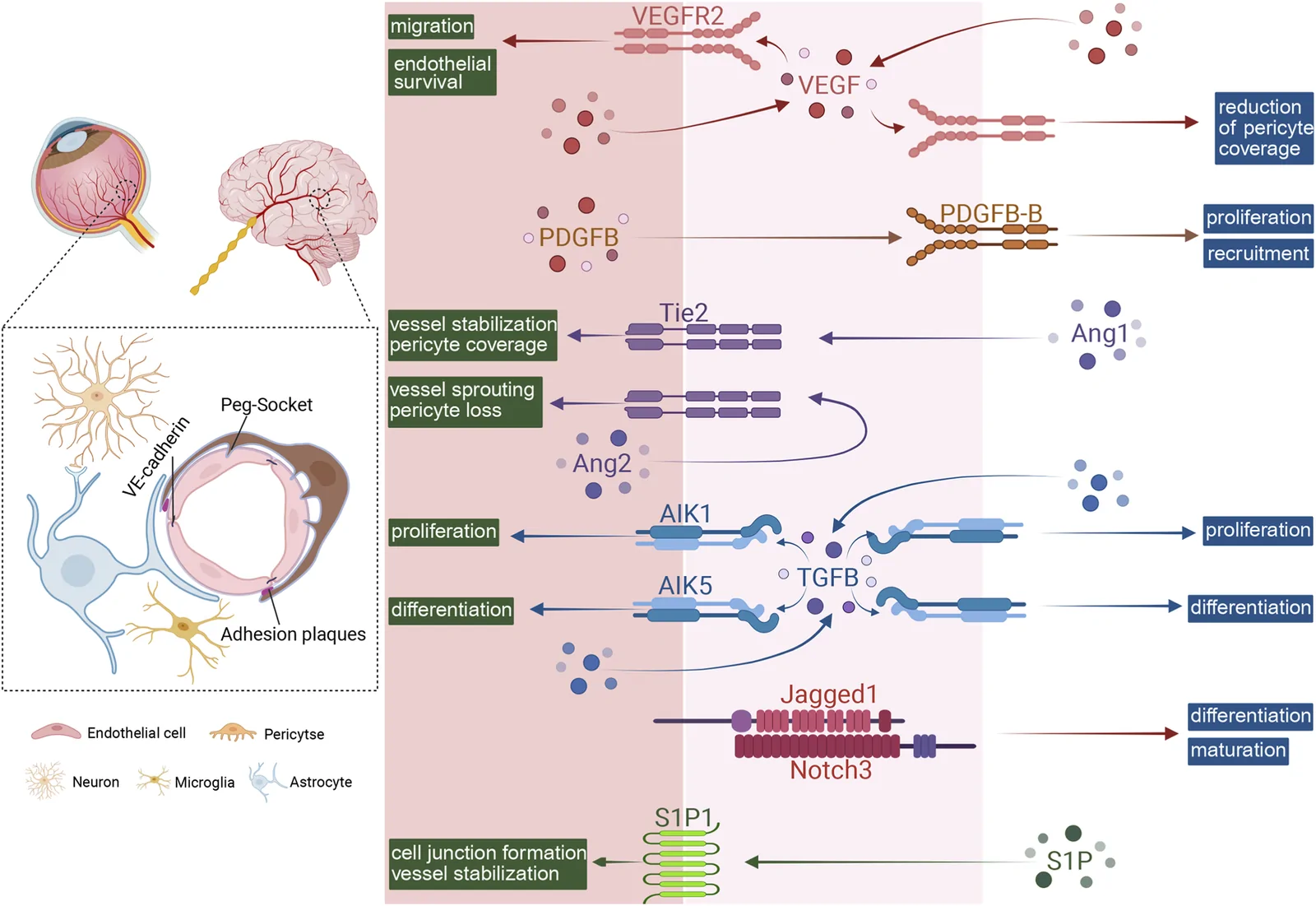
Pericytes govern vascular barrier homeostasis through integrated structural and signaling interactions.

Pericytes serve as important regulatory components that integrate inflammatory and mechanical cues, facilitating bidirectional communication with immune cells and endothelial cells to regulate vascular barrier function (Figure 2) (Chagnot et al., 2026) (bioRxiv, 2026).

Pericyte crosstalk with the inflammatory microenvironment. Pro-inflammatory mediators such as IL-1β, TNF-α, and LPS induce activation of NF-κB and MAPK pathways within pericytes (Persidsky et al., 2016). This activation can trigger a signaling feedback loop in which pericytes secrete chemokines, including CCL2, CXCL1, CXCL8, and IL-6 and upregulate adhesion molecules including ICAM-1 and VCAM-1 (Dabravolski et al., 2023; Wahl et al., 2024). As a result, leukocyte adhesion and transmigration along the vascular wall are regulated. Concurrently, pericytes remodel the microarchitecture of the vessel wall by modulating their contractile state and protrusion orientation. These adaptations guide immune cells along defined low-resistance routes, facilitating immune cell trafficking while limiting disruption to overall barrier integrity (Girbl et al., 2018). Under transient or controlled inflammatory conditions, pericytes may counteract this loop by secreting factors such as Ang-1, TGF-β, and neurotrophic factors, contributing to endothelial junction stabilization and helping to maintain barrier integrity. In contrast, sustained or excessive inflammatory stimulation may lead to elevated NF-κB-mediated chemokine and MMP-9 expression (Dave et al., 2024). This is associated with basement membrane degradation, and pericyte detachment, thereby amplifying endothelial activation and barrier disruption.

Pericytes interact directly with tissue-resident and infiltrating immune cells, including macrophages and leukocytes, extending beyond chemokine-driven recruitment to engage in bidirectional signaling crosstalk (London et al., 2013). Pericytes communicate with perivascular macrophages *via* soluble factors and possibly through contact-dependent mechanisms (Zhu et al., 2017). In turn, activated macrophages secrete PDGF-BB and TGF-β, which modulate pericyte proliferation, contractility, and expression of adhesion molecules (Guijarro-Muñoz et al., 2014; Rangasamy et al., 2020). This crosstalk establishes a cytokine signaling loop in which pericyte-derived cytokines influence macrophage polarization (M1 *versus* M2), while macrophage-derived signals reciprocally modulate pericyte phenotype and their barrier-supportive functions (Li et al., 2021). Similarly, pericytes interact with recruited leukocytes (neutrophils, monocytes, lymphocytes) not only through adhesion molecules but also through secreted factors such as CXCL family members, contributing to a paracrine network that is involved in immune cell trafficking and vascular responses (Medina-Flores et al., 2023). Dysregulation of this pericyte-immune crosstalk contributes to chronic inflammation, barrier dysfunction, and disease progression in conditions such as diabetic retinopathy and tumor microenvironments (Min et al., 2024).

Mechanical cue transduction and crosstalk with inflammatory signaling. Pericytes mediate mechanical inputs such as hemodynamic shear stress and ECM stiffness and convert them into intracellular signals *via* Piezo1, YAP/TAZ, TRPC3, TRPM4/7, and adhesion G protein-coupled receptors (GPCRs) (Hsu et al., 2023). Mechanical stimulation activates RhoA/ROCK, YAP/TAZ, and Ca^2+^-dependent pathways, regulating pericyte contractility and secretory profile (Meng et al., 2018; Shah et al., 2025). ECM stiffening enhances pericyte sensitivity to inflammatory cytokines. Inflammatory signals can also alter pericyte mechanotransduction. This integrated crosstalk determines the net barrier outcome. Under persistent inflammation or aberrant mechanics, pericyte mechanoregulatory capacity is compromised. This is associated with exaggerated permeability responses and barrier destabilization (Lim et al., 2024).

Building upon these canonical pathways, recent studies have further expanded the repertoire of mechanosensing mechanisms in pericytes. Beyond Piezo1 and YAP/TAZ, additional ion channels and signaling systems have been implicated in pericyte mechanotransduction. In cerebral capillaries, TRPC3 channels mediate pressure-induced pericyte depolarization and capillary constriction and are implicated in the regulation of cerebral perfusion (Ferris et al., 2025). Similarly, in cardiac pericytes, TRPM4 and TRPM7 channels detect compressive stress during ischemic injury, and their inhibition has been shown to attenuate infarct size (Methner et al., 2025). Notably, emerging evidence indicates that pericytes can function as direct sensors of ECM stiffness. Utilizing a microphysiological capillary-on-a-chip model, recent work has demonstrated that perivascular cells mediate vascular responses to fibrotic ECM stiffening through NOTCH3-dependent signaling (Franca et al., 2025). In addition, GPCRs, particularly adhesion GPCRs such as ADGRG1, have been identified as force-responsive receptors capable of undergoing activation under mechanical stimulation (Scholz et al., 2023; Xiao et al., 2023), although their precise roles in pericyte mechanotransduction remain to be fully elucidated. Collectively, these findings highlight that pericytes integrate mechanical cues through a diverse and expanding array of mechanosensors extending well beyond Piezo1 and YAP/TAZ, with significant implications for understanding pericyte dysfunction in vascular pathologies such as diabetic retinopathy.

Pericytes as hubs integrating inflammatory and mechanical crosstalk. Pericytes thus function as both sensors of inflammatory and mechanical cues and important components involved in vascular barrier state transitions (Fazio et al., 2024). This multi-directional crosstalk establishes a dynamic signaling network that governs vascular barrier transitions during homeostasis and disease. In microvascular pathologies such as diabetic retinopathy, disruption of pericyte-mediated crosstalk with immune cells, endothelium, and ECM components contributes to vascular barrier dysfunction.

In summary, pericytes are not merely passive mural cells. Through structural stabilization, signal modulation, and regulation of molecular transport, they actively maintain and regulate microvascular barrier function (Bhattacharya et al., 2020). From a translational medicine perspective, therapeutic strategies aimed at protecting or restoring pericyte function may stabilize the BBB and BRB (Crouch et al., 2015). These strategies may restore barrier function not only by preserving physical coverage of microvessels but also by restoring paracrine signaling networks and suppressing pathological transcellular transport (Davis and Senger, 2005).

### 3.4 Pericyte-derived extracellular vesicles in barrier regulation

In addition to direct cell-cell contact and paracrine signaling, pericytes communicate with neighboring cells through extracellular vesicles (EVs), including exosomes. This mode of intercellular communication is particularly relevant to the regulation of BBB and BRB. It should be noted that the available evidence regarding the functional roles of pericyte-derived extracellular vesicles (EVs) in vascular barrier regulation remains relatively limited, with many proposed mechanisms primarily based on *in vitro* studies or correlative *in vivo* observations. Pericyte-derived EVs carry diverse bioactive cargo, such as microRNAs, proteins, and lipids, which can modulate endothelial barrier properties (Scholz et al., 2023). For instance, pericyte-derived exosomes have been reported to transfer functional microRNAs (e.g., miR-210 and miR-148a-3p) to endothelial cells, contributing to improved barrier integrity and endothelial function through modulation of intracellular signaling and metabolic processes (Zhang et al., 2023). Consistent with these observations, pericyte-derived EVs have been shown to enhance endothelial barrier properties and reduce permeability in experimental models of injury (Gao et al., 2023; Zhang et al., 2023). Under inflammatory or pathological conditions, however, EV cargo composition may be altered, potentially leading to barrier-disruptive effects. Although direct evidence from pericyte-derived EVs remains limited, studies in other vascular cell types have shown that EV-associated factors such as matrix metalloproteinase-9 (MMP-9) and VEGF can promote tight junction disassembly and increase vascular permeability (Huang et al., 2017). These findings suggest that the functional effects of EV-mediated signaling are context-dependent and influenced by the molecular cargo packaged under specific conditions.

Emerging evidence further indicates a link between cellular metabolic state and EV-mediated communication (Sheng et al., 2025). In diabetic or high-glucose conditions, exosomes derived from pericyte-like cells exhibit altered cargo profiles and have been associated with changes in endothelial inflammation, oxidative stress, and permeability (Nandeesh et al., 2026; Wu et al. 2024). More generally, metabolic alterations including shifts in glycolysis, lipid metabolism, and redox balance have been shown to regulate EV biogenesis and cargo composition, thereby influencing responses in recipient cells (Crewe, 2023; Wang et al., 2024). While these observations support a potential role for metabolic regulation of EV signaling, the underlying mechanisms in pericytes remain to be fully defined. Further functional validation using *in vivo* models with pericyte-specific genetic manipulation of EV biogenesis pathways will be necessary to establish causal relationships between pericyte-derived EVs and vascular barrier function. EV-mediated communication represents a long-range mode through which pericytes contribute to signaling crosstalk.

These mechanistic insights have primarily been derived from studies of CNS pericytes, which exhibit unique properties related to their tight integration with the neurogliovascular unit. The extent to which these findings generalize to barrier-forming pericytes in other organs requires further investigation.

## 4 Pericytes and barrier state transitions

### 4.1 Diseases centered on the BRB

#### 4.1.1 Diabetic retinopathy

**FIGURE 3:**
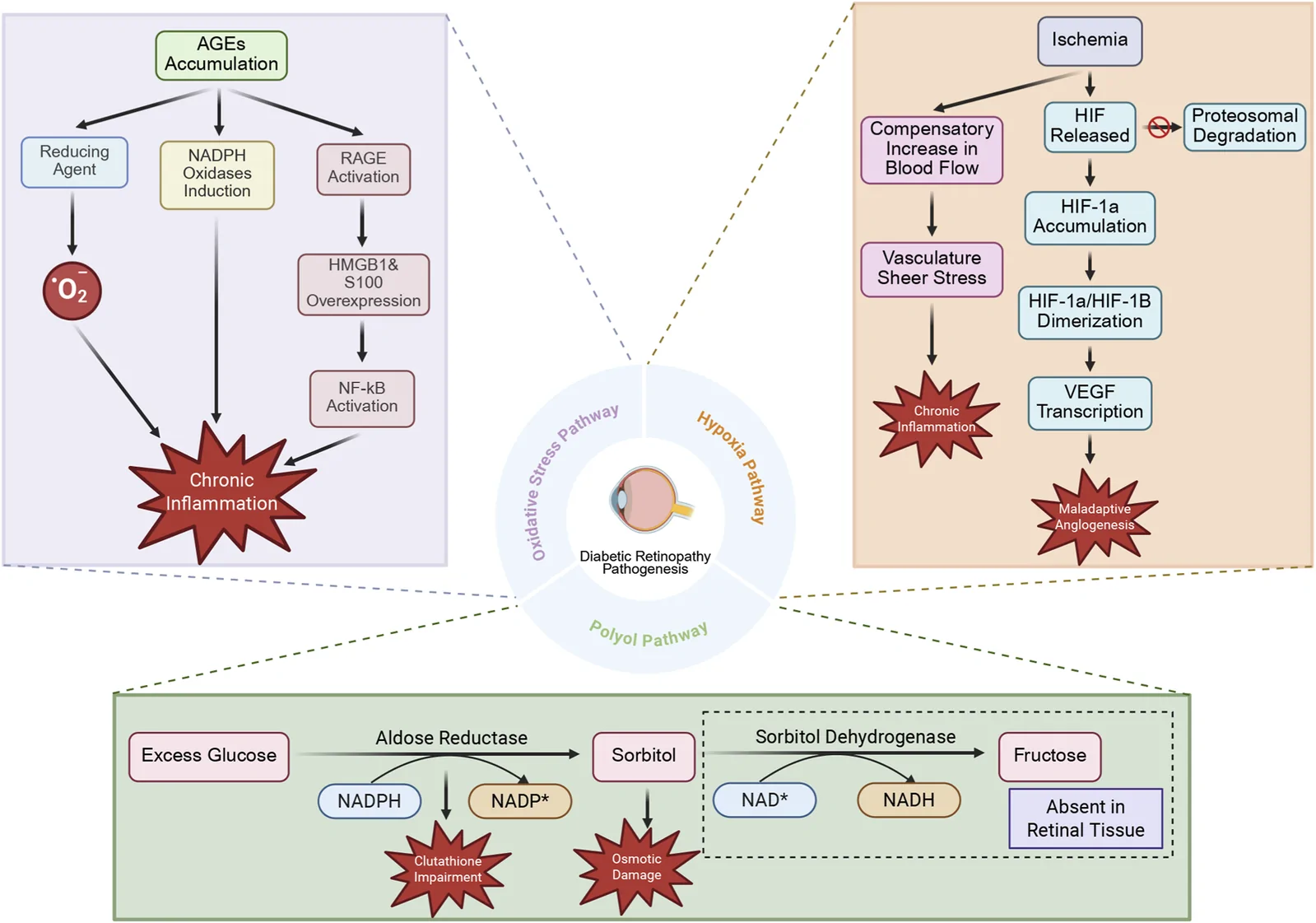
Convergent roles of pericyte dysfunction in blood-brain barrier disruption across neurological diseases.

DR is a common microvascular complication of diabetes and a leading cause of visual loss. Breakdown of BRB is a hallmark of early disease pathology. This dysfunction is closely linked to pericytes impairment, as pericytes are mural cells essential for maintaining vascular stability and integrity. Pericyte loss is often observed in the early stages of DR and may contribute to subsequent BRB dysfunction and capillary abnormalities, although this process likely occurs in conjunction with other cellular and systemic alterations during disease progression (Hammes et al., 2002). BRB dysfunction in DR is a multifactorial process involving not only pericyte loss but also endothelial dysfunction, inflammatory activation, glial cell reactivity, and systemic metabolic disturbances. The relative contribution of each factor varies across disease stages and among individual patients. Numerous clinicopathological studies have consistently reported a significant reduction in pericyte density within retinal capillaries of patients with DR (Figure 3). Retinal flat-mount analyses further reveal increased pericyte apoptosis in diabetic retinas compared with nondiabetic controls (Li et al., 1997). This was accompanied by TUNEL-positive cells and characteristic pericyte “ghosts,” indicating that apoptosis represents a major mechanism underlying pericyte loss (Robison et al., 1991).

Under chronic hyperglycemic conditions, several interrelated metabolic pathways contribute to pericyte injury and microvascular dysfunction (Brownlee, 2005). An important mechanism is the activation of the polyol pathway, in which excess intracellular glucose is converted into sorbitol *via* aldose reductase (Lorenzi, 2007). This process consumes NADPH and reduces cellular antioxidant capacity by impairing glutathione regeneration, thereby weakening the ability of cells to neutralize reactive oxygen species (ROS). Concurrently, intracellular sorbitol accumulation induces osmotic stress and cellular swelling, further compromising pericyte integrity and function. Hyperglycemia also accelerates the formation of advanced glycation end-products (AGEs). Binding of AGEs to their receptor, RAGE, activates NADPH oxidase and enhances ROS generation while inducing inflammatory mediators such as HMGB1 and S100 proteins (Kowluru et al., 2010). These events stimulate downstream inflammatory signaling pathways, such as NF-κB. Activation of these pathways establishes a state of chronic inflammation and persistent oxidative stress within the retinal microvasculature. Sustained oxidative stress can disrupt mitochondrial function and trigger pro-apoptotic signaling pathways, including Bax/Bcl-2 imbalance and caspase activation, thereby promoting pericyte apoptosis (Beltramo and Porta, 2013).

Recent studies have further identified mitochondrial redox regulation as an important determinant of pericyte survival. For instance, downregulation of glutaredoxin 2 (Grx2) under hyperglycemic conditions impairs mitochondrial function in pericytes, resulting not only in increased apoptosis but also in reduced pericyte-endothelial interaction and increased vascular permeability. These findings highlight that mitochondrial dysfunction directly contributes to impaired intercellular communication within the retinal microvasculature. Furthermore, hyperglycemia activates the protein kinase C (PKC) signaling pathway, which contributes to microvascular dysfunction. PKC activation affects vascular permeability, extracellular matrix deposition, and hemodynamic regulation while promoting the expression of inflammatory cytokines and angiogenic factors. These alterations aggravate endothelial dysfunction and contribute to basement membrane thickening. As capillary nonperfusion progresses, retinal hypoxia is associated with stabilization of hypoxia-inducible factor-1α (HIF-1α), which contributes to the transcription of angiogenic genes such as VEGF (Stitt et al., 2016). Although VEGF upregulation initially reflects a compensatory response to retinal ischemia, persistent overexpression promotes pathological angiogenesis and further increases vascular permeability.

Importantly, these metabolic, oxidative, and inflammatory disturbances not only induce pericyte injury but also disrupt pericyte-mediated microenvironmental signaling crosstalk. Under physiological conditions, pericytes engage in dynamic bidirectional communication with endothelial cells through signaling pathways such as PDGF-B/PDGFR-β and Ang-1/Tie2, thereby maintaining endothelial stability and vascular homeostasis (Park et al., 2017). However, under diabetic conditions, impairment or loss of pericytes is associated with disruption of vascular signaling interactions, often accompanied by endothelial destabilization, increased vascular permeability, and dysregulated extracellular matrix remodeling. In addition, hyperglycemia-induced inflammatory signaling enhances aberrant crosstalk between pericytes and other microenvironmental components, including immune mediators and endothelial-derived factors, further amplifying vascular dysfunction. This disruption of intercellular communication contributes to increased transendothelial transport, tight junction impairment, and progressive BRB breakdown.

Emerging evidence points to extracellular vesicles, especially exosomes, as important mediators of intercellular communication in DR (Xu et al., 2022). These vesicles carry noncoding RNAs (such as microRNAs and long noncoding RNAs) whose altered levels have been linked to endothelial dysfunction, inflammation, and angiogenesis (Xia et al., 2023). This adds a new layer to pericyte-associated signaling crosstalk. In addition, advances in single-cell and spatial transcriptomics have revealed complex multicellular communication networks in DR (Mrowicka et al., 2024). Pericytes, endothelial cells, immune cells, and glial cells all engage in dynamic, context-dependent interactions (Xiao and Xu, 2024). Together, these findings support a view of DR as a disease caused by disrupted multicellular crosstalk, not just by isolated cellular defects.

From a clinical perspective, modulation of pericyte-mediated signaling pathways has been proposed as a potential therapeutic avenue, although its clinical feasibility remains to be established. Current anti-VEGF therapies partially restore vascular homeostasis but do not directly address pericyte loss or dysfunction. Therefore, novel approaches have been explored to preserve pericyte survival, support pericyte-endothelial communication, or modulate oxidative stress or mitochondrial dysfunction, although most remain at the preclinical or early translational stage (Warmke et al., 2021). In addition, exosome-based therapies and cell-based strategies to modulate or support pericyte function are being investigated as emerging experimental approaches (Dubrac et al., 2018). These strategies highlight the therapeutic potential of restoring microenvironmental signaling crosstalk rather than targeting single pathways alone.

Collectively, these findings suggest that DR progression is closely associated with disrupted pericyte-mediated signaling crosstalk, leading to imbalance in endothelial stability, inflammatory responses, and microenvironmental homeostasis (Rangasamy ettt al., 2020). This perspective supports a paradigm shift in understanding DR as a disease driven by multicellular network dysfunction and underscores the importance of targeting intercellular communication for future therapeutic development. In addition to pericyte injury, endothelial metabolic dysfunction, Müller cell activation, and immune-mediated inflammation also contribute to the breakdown of the blood-retinal barrier, highlighting the multicellular nature of diabetic retinopathy.

#### 4.1.2 Retinal vein occlusion

In contrast to the progressive microvascular injury driven by chronic metabolic disturbances in diabetic retinopathy, disruption of the BRB in RVO typically occurs acutely and in a spatially restricted manner, suggesting that hemodynamic stress plays a major role in barrier dysfunction (Hayreh, 1971). Under physiological conditions, pericytes are important regulators of retinal microvascular homeostasis (Winkler et al., 2010). By modulating capillary tone and buffering local fluctuations in blood flow, pericytes help maintain endothelial stability and preserve a low permeability barrier phenotype that is essential for BRB integrity (Abbott et al., 2006). Following venous occlusion, the abrupt elevation of venous pressure and alterations in shear stress impose substantial mechanical strain on the retinal microvascular wall (Rehak and Rehak, 2008). These hemodynamic disturbances may impair the functional interaction between pericytes and endothelial cells, thereby weakening the stabilizing signals that normally support endothelial barrier properties (Beltramo and Porta, 2013). In vascular segments where pericyte coverage is reduced or their regulatory capacity is compromised, endothelial cells may become more susceptible to permeability-inducing stimuli. Such conditions can promote pro-permeability responses, including activation of VEGF signaling, cytoskeletal remodeling, and increased vesicle-mediated transcellular transport (Andreoli and Miller, 2007).

Alterations in endothelial barrier function may occur before extensive structural disruption of tight junctions becomes evident. In addition to hemodynamic stress, venous obstruction also induces local retinal ischemia, which stabilizes HIF-1α and promotes VEGF expression, further increasing vascular permeability and contributing to retinal edema (Haydinger et al., 2023). Within this context, impaired pericyte-mediated regulation may amplify endothelial responses to hemodynamic and hypoxic stimuli. Given the heterogeneous distribution and functional status of pericytes within the retinal microvasculature, this regulatory imbalance may occur preferentially in specific vascular segments, potentially explaining the characteristic focal vascular leakage observed clinically in RVO(Rehak and Rehak, 2008). Recent advances further suggest that pericytes actively participate in mechanotransductive signaling, translating hemodynamic forces into biochemical responses that regulate endothelial behavior. Emerging evidence indicates that alterations in mechanosensitive pathways, including calcium signaling and cytoskeletal dynamics, may impair pericyte-endothelial communication under conditions of acute vascular stress. In addition, extracellular vesicles and inflammatory mediators released in response to hemodynamic injury may further modulate intercellular signaling, adding an additional layer of microenvironmental crosstalk in RVO. From the perspective of the pericyte-endothelial functional unit, BRB dysfunction in RVO may therefore reflect a state of acute barrier dysregulation in which pericyte regulatory capacity is insufficient to counteract hemodynamic and ischemic stress. This concept is supported by recent imaging and single-cell analyses. These analyses indicate that vascular dysfunction in RVO is associated with dynamic and spatially heterogeneous alterations in intercellular communication networks, rather than uniform structural damage. From a translational perspective, current therapies for RVO, particularly anti-VEGF agents, effectively reduce vascular leakage. However, they do not directly address pericyte dysfunction or impaired intercellular communication. Therefore, emerging strategies are being explored to overcome these limitations. These include approaches aimed at stabilizing pericyte–endothelial interactions, modulating mechanotransduction pathways, and targeting inflammatory signaling as well as extracellular vesicle-mediated communication. In addition, strategies that preserve pericyte contractile balance may help optimize vascular tone regulation. Such approaches could limit both excessive vascular leakage and ischemic injury. Collectively, these strategies highlight the therapeutic potential of restoring microenvironmental signaling crosstalk as a complementary paradigm in the treatment of RVO(Campochiaro, 2015; Glacet-Bernard et al., 2011).

RVO is highly context-dependent and remains incompletely understood. Although pericyte dysfunction is associated with increased vascular permeability, it is still debated whether their response to acute hemodynamic stress is purely pathological or confers a degree of adaptive benefit. For instance, transient pericyte-mediated vasoconstriction may help stabilize capillary pressure and limit fluid extravasation, whereas sustained contraction could exacerbate ischemia and contribute to tissue injury. This dual functionality suggests that pericytes operate within a dynamic equilibrium between protective and detrimental effects. Defining the temporal thresholds and underlying molecular mechanisms governing this functional transition may facilitate the development of targeted therapeutic strategies.

#### 4.1.3 Retinopathy of prematurity

ROP arises during retinal development and is characterized not by disruption of a mature BRB but by abnormal vascular growth and maturation, characterized not by disruption of a mature BRB but by interrupted and aberrant vascular growth and maturation (Sapieha et al., 2010; Strube and Wright, 2022). Exposure of the preterm retina to non-physiological oxygen levels disrupts vascular sprouting, leading to avascular zones; subsequently, the relatively hypoxic retinal regions induce excessive pro-angiogenic signaling, driving pathological neovascularization (Rashidian et al., 2024). This pathophysiological sequence has been systematically recapitulated in OIR models (Smith et al., 1994). Early hyperoxia suppresses physiological vascular growth, delaying vessel maturation and barrier formation (Connor et al., 2009). Subsequent relative hypoxia reactivates angiogenic programs, but newly formed vessels emerge in the absence of sufficient maturation and stabilizing signals (Stahl et al., 2010). This biphasic process has been well established in OIR models.

OIR models demonstrate that early hyperoxic exposure interferes with vascular maturation. Prior to full establishment of the vascular barrier, hyperoxia inhibits vessel growth. Signaling pathways that normally rely on hypoxic conditions to drive endothelial differentiation toward a barrier phenotype are suppressed. These pathways include Wnt/β-catenin and other endothelial maturation pathways (Stenman et al., 2008). As a result, endothelial cells remain in a relatively immature state, exhibiting incomplete transcellular transport regulation and insufficient intercellular junctional differentiation. During the subsequent relative hypoxia phase, vascular regeneration is reinitiated, yet pro-angiogenic signals and barrier-stabilizing cues are temporally desynchronized (Ratcliffe, 2013). Under hypoxic stress, rapid endothelial expansion preferentially activates permeability-enhancing programs, such as VEGF-driven cytoskeletal remodeling and vesicular transport, while signals maintaining barrier integrity fail to fully recover (Wakasugi et al., 2024). It is important that delayed pericyte recruitment and impaired pericyte-endothelial signaling further exacerbate this imbalance. Under physiological conditions, pericytes provide stabilizing cues that promote endothelial maturation and barrier integrity. In ROP, however, insufficient or mistimed pericyte coverage disrupts this signaling crosstalk, preventing nascent vessels from acquiring a mature, low-permeability phenotype. Recent studies also suggest that altered metabolic states and impaired growth factor signaling, including insulin-like growth factor-1 (IGF-1), may further compromise pericyte-endothelial communication during retinal vascular development.

From a translational perspective, current therapeutic strategies, including anti-VEGF agents, have demonstrated efficacy in reducing pathological neovascularization. However, their effects on vascular maturation and long-term neurovascular development remain uncertain. Increasing attention is therefore being directed toward strategies that restore physiological vascular development, such as optimizing oxygen management, supplementing IGF-1 to support normal vascular growth, and modulating Wnt signaling to promote barrier maturation. In addition, approaches aimed at enhancing pericyte recruitment and stabilizing pericyte-endothelial interactions may provide complementary benefits by improving vascular integrity and reducing leakage.

Overall, ROP represents a condition of delayed or incomplete BRB maturation, with its pathological hallmark being functional immaturity of the vascular barrier rather than secondary disruption of mature vessels (Sabri et al., 2022). Early interventions aimed at supporting pericyte recruitment and promoting barrier maturation may reduce pathological neovascularization and leakage, offering potential therapeutic strategies for ROP(Dammann et al., 2023).

Despite these advances, a key unresolved issue is whether vascular abnormalities in ROP primarily reflect delayed maturation or represent a state of pathological developmental dysregulation. Addressing this question will require further investigation into the integration of developmental signaling, metabolic regulation, and intercellular communication. A deeper understanding of these processes may facilitate the development of stage-specific therapeutic strategies aimed at restoring microenvironmental signaling during retinal vascular development.

### 4.2 Pericytes in blood-brain barrier diseases

#### 4.2.1 Ischemic stroke

Ischemic stroke is triggered by the acute occlusion of a cerebral artery, leading to rapid energy depletion in the affected brain tissue (Dirnagl et al., 1999). ATP exhaustion disrupts ionic homeostasis and induces cellular depolarization, initiating a cascade of metabolic and inflammatory responses that ultimately compromise BBB function and contribute to secondary brain injury (Iadecola and Anrather, 2011). An important event in ischemic stroke is the disruption of the neurovascular unit, of which pericytes are an important component. Under physiological conditions, pericytes rely heavily on mitochondrial oxidative metabolism to sustain contractile regulation and barrier-supporting functions, rendering pericytes highly susceptible to ischemia and oxidative stress. Animal models have demonstrated that pericytes exhibit calcium dysregulation, mitochondrial dysfunction, and phenotypic activation within minutes of ischemic onset (Kisler et al., 2017). Some cells undergo apoptosis or functional loss within an hour, preceding overt endothelial damage and BBB leakage. This temporal sequence suggests that pericyte dysfunction may represent an early contributor to of barrier disruption rather than merely a secondary consequence.

As ischemia and subsequent reperfusion progress, pericyte-endothelial interactions are further impaired. Some pericytes detach from the vessel wall, diminishing their support of endothelial homeostasis and leading to increased transcellular transport and abnormal tight junction distribution (Yang and Rosenberg, 2011). Concurrently, ischemia stimulates pericytes and other neurovascular unit cells to release proteases, including MMPs, leading to basement membrane degradation and amplification of barrier disruption (Rosenberg and Yang, 2007). Sustained hypoxia-induced pericyte constriction has been implicated in capillary no-reflow, though the relative contribution of pericyte-mediated *versus* arteriolar smooth muscle-mediated mechanisms remains debated (Yemisci et al., 2009). This perfusion deficit not only limits the restoration of oxygen and nutrient supply but also exacerbates local metabolic stress, further destabilizing the BBB and establishing a vicious cycle between impaired perfusion and barrier dysfunction. Recent studies further indicate pericytes participate in inflammatory signaling and microenvironmental crosstalk following stroke. Single-cell analyses have revealed dynamic interactions among pericytes, endothelial cells, and infiltrating immune cells, indicating that pericytes actively participate in post-ischemic inflammatory responses and vascular remodeling (Li et al., 2025). Moreover, alterations in pericyte metabolic state and redox signaling have been shown to influence their ability to maintain endothelial stability and barrier function (Garcia-Bonilla et al., 2024).

Although intravenous thrombolysis and mechanical thrombectomy can restore large-vessel patency, microcirculatory perfusion and BBB integrity often fail to recover in parallel (Sun et al., 2024). Therefore, emerging therapeutic strategies are increasingly focused on targeting pericyte function, including modulation of pericyte contraction, inhibition of MMP activity, and attenuation of oxidative stress (Ogunshola and Tsao, 2024). In addition, approaches aimed at restoring pericyte–endothelial communication and regulating post-ischemic inflammation may enhance microvascular reperfusion and reduce secondary brain injury (Seyedaghamiri et al., 2024).

Collectively, these findings suggest that ischemic stroke can be conceptualized as a disorder of disrupted pericyte-mediated signaling crosstalk, in which early pericyte dysfunction drives BBB breakdown, microvascular failure, and neuroinflammation (Denny-Brown and Meyer, 1957). Targeting these intercellular signaling networks may provide a complementary strategy to improve outcomes beyond conventional reperfusion therapies. BBB disruption in ischemic stroke reflects multicellular dysfunction of pericytes, endothelial cells, astrocytes, and infiltrating immune cells, rather than pericyte loss alone.

#### 4.2.2 Alzheimer’s disease

AD is a neurodegenerative disorder characterized by progressive cognitive decline and represents the leading cause of dementia in the elderly (De Strooper and Karran, 2016; Hardy and Selkoe, 2002). Increasing evidence indicates that, in addition to neuronal pathology, dysfunction of the BBB constitutes an important early pathological event in AD, with its severity closely correlating with cognitive impairment (Nation et al., 2019; Sweeney et al., 2018). Under physiological conditions, the BBB relies on highly specialized endothelial cells, intact tight junctions, and the regulation by pericytes and astrocytes to maintain selective permeability to blood-borne molecules (Iadecola, 2017). This highly selective barrier is essential for the long term stability of neuronal function.

In the brains of AD patients, pathological stimuli such as β-amyloid deposition, oxidative stress, and chronic low-grade inflammation disrupt neurovascular unit homeostasis, often associated with increased BBB permeability (Zlokovic, 2004; Zlokovic, 2011). Multiple studies have shown that BBB dysfunction can be detected even before overt tight junction disruption, suggesting that barrier imbalance precedes structural damage (Nation et al., 2019). Recently, the role of pericytes in AD-associated BBB dysfunction has garnered significant attention. Investigations have revealed reduced pericyte coverage of cerebral microvessels, elevated levels of soluble PDGFRβ in cerebrospinal fluid, and strong associations with BBB leakage and cognitive decline (Halliday et al., 2016). Vascular β-amyloid deposits exert chronic toxic effects on pericytes, compromising their ability to maintain endothelial barrier properties (Sagare et al., 2013). Dynamic contrast-enhanced MRI (DCE-MRI) studies have confirmed that early BBB leakage can be detected in AD, particularly in the hippocampus and entorhinal cortex, regions closely linked to cognitive function (Montagne et al., 2015).

Therapeutic strategies targeting neurovascular dysfunction are gaining increasing attention. While current approaches primarily focus on Aβ clearance, emerging strategies aim to preserve BBB integrity and restore NVU signaling. These include modulation of pericyte-associated pathways, such as PDGF-B/PDGFRβ signaling, inhibition of ApoE4-mediated inflammatory responses, and targeting vascular signaling pathways including VEGF and Notch3 (Vrillon et al., 2025). In addition, interventions aimed at reducing neuroinflammation and oxidative stress may indirectly improve pericyte function and intercellular communication (Korte et al., 2024). Collectively, these findings suggest that AD can be conceptualized as a disorder of disrupted pericyte-mediated signaling crosstalk, in which early pericyte dysfunction contributes to BBB dysfunction, neuroinflammation, and progressive cognitive decline (Miners et al., 2019; Yang et al., 2024). Targeting these intercellular signaling networks may provide a complementary strategy for slowing disease progression beyond traditional amyloid-centered approaches.

#### 4.2.3 Multiple sclerosis

Multiple sclerosis (MS) is a chronic inflammatory disorder of the central nervous system, primarily contribute to by aberrant activation of the immune system and infiltration of immune cells into the brain parenchyma (Lassmann, 2018; Ransohoff, 2016). BBB disruption is not merely a secondary consequence of tissue injury. Instead, it constitutes a prerequisite for the initiation of immune-mediated pathological processes (Alvarez et al., 2011). Extensive imaging evidence indicates that focal increases in BBB permeability precede the formation of demyelinating lesions and clinical relapses (Minagar and Alexander, 2003). This phenomenon has been consistently observed in both MS patients and experimental autoimmune encephalomyelitis (EAE) models, highlighting BBB compromise as an early event in neuroinflammation (Ortiz et al., 2013).

Within this unique inflammatory milieu, pericytes undergo functional reprogramming, acquiring pronounced immunoregulatory capabilities (Jansson et al., 2014). Activated pericytes upregulate adhesion molecules such as ICAM-1 and VCAM-1, enabling direct participation in leukocyte adhesion to the vascular wall and subsequent transmigration into the brain parenchyma (Engelhardt and Ransohoff, 2012). Pericytes modulate the trafficking of immune cells across the BBB, contributing to an additional layer of immune regulation beyond the endothelium (Stark et al., 2013). Persistent inflammatory signaling drives pericytes toward a profibrotic phenotype, accompanied by elevated MMPs activity and collagen deposition, ultimately resulting in perivascular fibrosis and basement membrane remodeling (Rosenberg, 2009). This vascular remodeling not only impedes nutrient exchange and restricts the migration of reparative cells but also creates a microenvironment hostile to remyelination and axonal repair. Concurrently, the diminished capacity of pericytes to regulate vascular tone exacerbates local hypoperfusion, further amplifying neuronal and myelin injury. Recent studies have further highlighted the role of pericytes in coordinating microenvironmental signaling crosstalk in MS. Single-cell analyses have revealed dynamic interactions between pericytes, endothelial cells, and infiltrating immune cells, with enrichment of inflammatory and immune-regulatory pathways.

Pericyte dysfunction in MS involves multiple key signaling pathways. Attenuation of PDGF-B/PDGFR-β signaling limits pericyte recruitment, disruption of the Notch pathway compromises vascular stability, and aberrant TGF-β signaling promotes both fibrotic transformation and immune activation (Liu et al., 2010; Walshe et al., 2009). Under conditions of oxidative stress and excessive ROS, activated pericytes may experience mitochondrial dysfunction, further weakening support for endothelial integrity and increasing BBB permeability (Ortiz et al., 2013). Current MS therapies primarily target immune modulation but do not directly address neurovascular dysfunction (Yu et al., 2025). Emerging strategies are in the context, therefore beginning to consider the vascular component of the disease. Approaches aimed at stabilizing BBB integrity, modulating pericyte activation, and inhibiting MMP-mediated matrix remodeling are under investigation (Kaushik et al., 2021). In addition, targeting inflammatory signaling pathways that mediate pericyte-immune cell interactions may provide a novel avenue for limiting immune cell infiltration and promoting tissue repair (King et al., 2024).

In summary, pericytes in MS serve not only structural support roles but also act as dynamic regulators of BBB function and immune cell infiltration (Herman and Jacobson, 1988). Their dysfunction constitutes both a prerequisite for inflammatory infiltration and a pivotal contribute to demyelinating lesion propagation and neural injury (Iacobaeus et al., 2017; Şekerdağ-Kılıç et al., 2025). Targeting pericyte-mediated signaling crosstalk may in the context, therefore offer a complementary strategy to existing immunotherapies.

**FIGURE 4:**
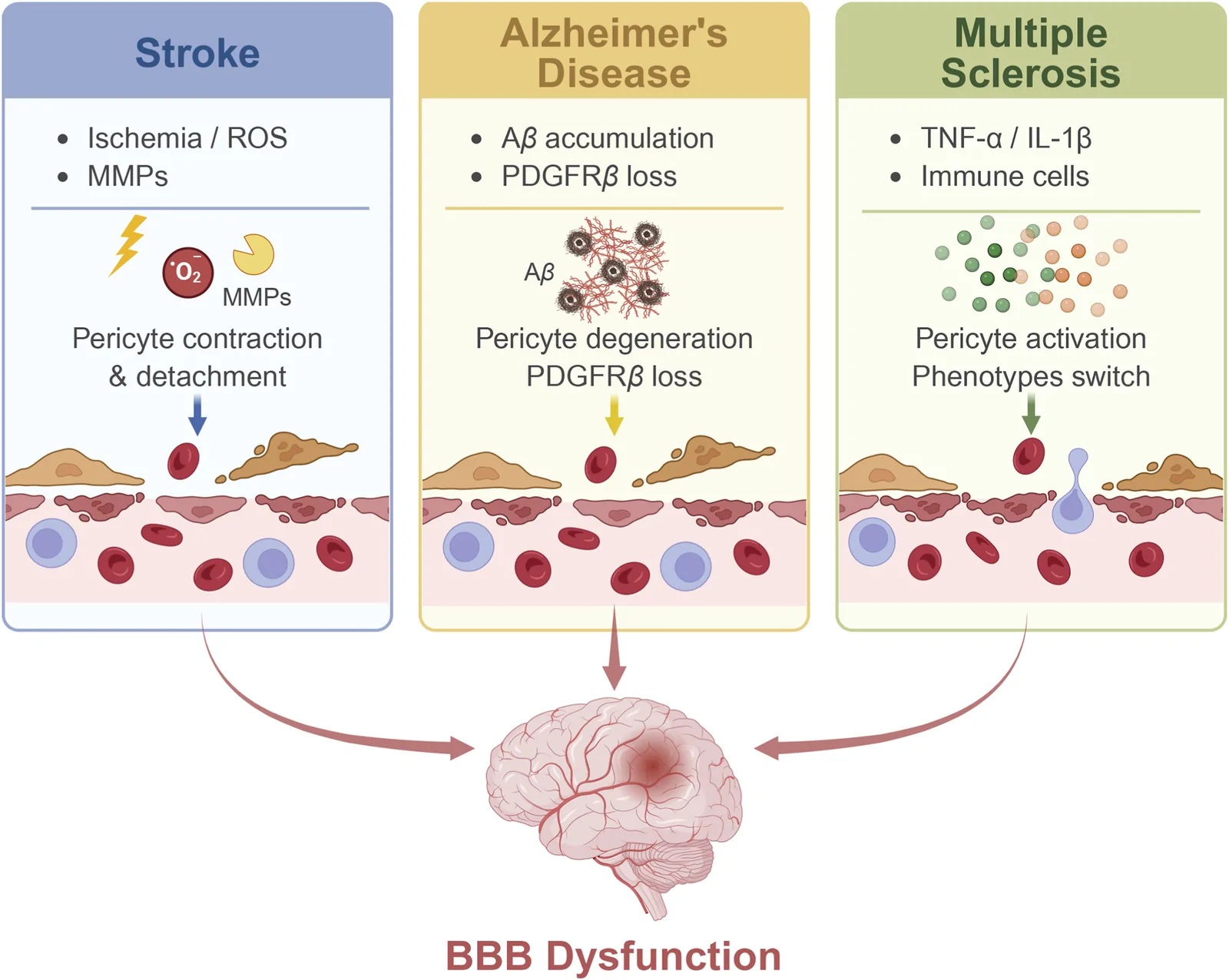
Pericyte dysfunction as a convergent hub leading to BBB disruption across distinct cerebrovascular and neurodegenerative diseases.

Overall, pericytes play important and multifaceted role in maintaining BBB integrity across a spectrum of cerebrovascular diseases (Figure 4). In ischemic stroke, early pericyte dysfunction characterized by metabolic failure, contraction, and detachment acts as important contributors to BBB disruption and microcirculatory impairment (Yemisci et al., 2009). In AD, progressive loss of pericyte coverage and functional integrity drives early BBB disruption, thereby accelerating neurodegenerative processes and cognitive decline.By contrast, in multiple sclerosis, inflammation-induced phenotypic reprogramming of pericytes is associated with enhanced immune cell infiltration and promotes fibrotic remodeling, ultimately exacerbating barrier dysfunction. Despite the distinct pathological triggers underlying these conditions, a common theme emerges in which pericyte dysfunction represents a convergent mechanism leading to BBB impairment (ElAli et al., 2014; Garcia-Gallardo and Campbell, 2025). Therefore, targeting pericyte survival, functional stability, and signaling pathways may provide a promising therapeutic strategy for preserving neurovascular integrity and mitigating disease progression in diverse cerebrovascular and neurodegenerative disorders. In multiple sclerosis, pericyte activation occurs in parallel with endothelial adhesion molecule upregulation and astrocyte-mediated inflammatory signaling, collectively facilitating immune cell infiltration.

Despite being discussed in separate disease contexts, BBB and BRB share a highly conserved structural and signaling architecture. In both systems, pericyte–endothelial interactions form the core regulatory unit governing barrier integrity, mediated through common signaling pathways including PDGF-B/PDGFRβ, TGF-β, VEGF, Ang-1/Tie2, and Notch signaling. Moreover, both barriers rely on tightly regulated junctional complexes, basement membrane remodeling, and finely tuned neurovascular or retinal vascular coupling to maintain homeostasis.

Importantly, disease-associated barrier disruption in both the BBB and BRB converges on a limited number of common pathological modules, including pericyte loss or dysfunction, endothelial activation, inflammatory signaling amplification, and metabolic or oxidative stress. These shared mechanisms suggest that BBB and BRB should be conceptualized as organ-specific manifestations of a unified microvascular barrier program rather than independent regulatory systems.

### 4.3 Comparative perspectives on BBB and BRB disorders and shared insights

Despite the structural and functional similarities between the BBB and BRB, increasing evidence highlights important context-specific differences including variations in pericyte density, metabolic demands, and immune microenvironment composition across tissues (Chen et al., 2019; Fan and Li, 2025; Frank et al., 1990; van Noorden et al., 2024). These differences may influence the relative contribution of individual vascular and parenchymal cell types to barrier dysfunction in disease settings (Uemura et al., 2021).

Under pathological conditions, both BBB and BRB disorders are characterized by increased vascular permeability and immune cell infiltration; However, the initiating and dominant cellular drivers vary substantially across disease contexts. Rather than acting as a single initiating factor, pericyte dysfunction is more appropriately considered one component of a broader multicellular network disturbance that includes endothelial activation, astrocytic dysregulation, immune cell recruitment, and systemic metabolic or inflammatory stress (D'Esposito et al., 2025). Similarly, similar pericyte-associated alterations may correlate with different vascular phenotypes across tissues, reflecting the influence of local endothelial signaling, extracellular matrix composition, and inflammatory milieu rather than pericyte dysfunction alone Disease kinetics and reversibility also differ between the BRB and BBB. The BRB may exhibit relatively higher regenerative capacity and structural plasticity, whereas the BBB is often less permissive to repair after injury. he BRB also provides experimental advantages, including accessibility for imaging and real-time assessment of microvascular dynamics; (Sotani et al., 2025).

**FIGURE 5:**
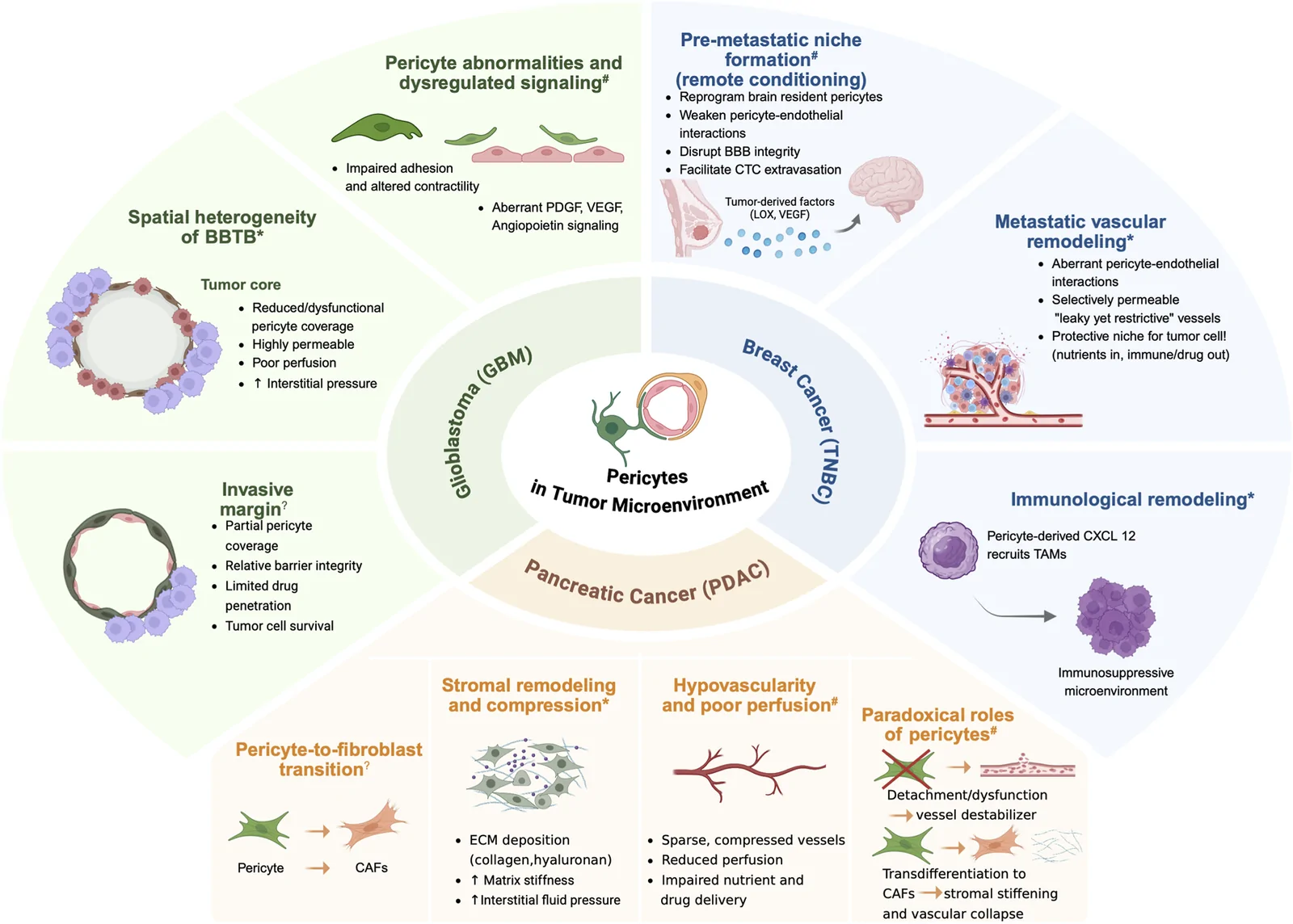
Pericytes in the tumor microenvironment paradoxically regulate blood tumor barrier integrity and pre-metastatic niche formation through metabolic reprogramming, dysfunctional endothelium interaction, and transdifferentiation into fibroblasts. Interactions depicted in this figure are annotated according to the level of evidence: strong experimental evidence is indicated by an asterisk (*), well-supported mechanistic frameworks are marked with a hash symbol (#), and emerging or hypothetical concepts are indicated by a question mark (?).

Collectively, current evidence supports a unifying concept in which BBB and BRB dysfunction arise from emergent properties of multicellular vascular units, rather than from dysfunction of any single-cell type alone (Kawoos et al., 2021; Lendahl et al., 2019). Within this system, pericytes represent important regulatory nodes that modulate vascular stability, but their functional impact is tightly dependent on interactions with endothelial cells, astrocytes, immune cells, and systemic factors. Finally, these context-dependent interactions become further amplified in tumor-associated vasculature, where dysregulation of multiple cell types contributes to heterogeneous vascular phenotypes, including hyperpermeability and perfusion abnormalities (Figure 5).

### 4.4 Pericytes in tumor microenvironment

It is important to distinguish tumor-associated pericytes from cancer-associated fibroblasts (CAFs), as these 2 cell populations share overlapping marker expression and functional properties but represent distinct perivascular lineages with important differences (Xian et al., 2006). Both pericytes and CAFs can express α-SMA, PDGFRα/β, and fibroblast activation protein (FAP), and both populations contribute to extracellular matrix remodeling, immune modulation, and therapeutic resistance within the tumor microenvironment (Akanda et al., 2023). However, pericytes are defined by their anatomical location embedded within the vascular basement membrane and maintaining direct, contact-dependent interactions with endothelial cells whereas CAFs are more broadly distributed throughout the tumor stroma and are not obligatorily associated with blood vessels. Functionally, pericytes primarily regulate vascular stability, barrier function, and endothelial survival, while CAFs are more prominently involved in ECM production, direct tumor cell invasion support, and paracrine signaling to cancer cells that promotes proliferation and stemness (Siedlecki et al., 2017). During tumor progression, pericyte detachment from the vasculature and potential transdifferentiation into fibroblast-like cells can further blur the distinction between these populations, and in some tumor types, a significant fraction of α-SMA + stromal cells may derive from detached pericytes. Conversely, certain CAF subpopulations may express pericyte markers without assuming a perivascular location. Improved lineage tracing tools, multi-marker profiling strategies, and spatial transcriptomic approaches are needed to rigorously resolve the identity, origin, and functional contributions of pericyte *versus* CAF populations within the tumor microenvironment.

#### 4.4.1 Glioblastoma: pericyte-driven vascular abnormalities and barrier compromise

Glioblastoma (GBM) exhibits a profoundly abnormal vascular system that departs from the tightly regulated architecture of the BBB. Rather than a uniform disruption, GBM establishes a spatially heterogeneous blood brain tumor barrier (BBTB), in which tumor-associated pericytes (TAPs) play a important role in shaping regional vascular function (Chang et al., 2017; Pombero et al., 2023).

As discussed above, GBM-associated pericytes arise from both resident and tumor-derived sources and display significant phenotypic abnormalities, including impaired endothelial adhesion and altered contractility (Salazar-Saura et al., 2024). In this context, dysregulated pericyte-endothelial interactions driven by aberrant PDGF, VEGF, and Angiopoietin signaling do not simply destabilize vessels but generate a functionally heterogeneous vascular network with variable permeability and perfusion (Di Tacchio et al., 2019).

This heterogeneity is particularly evident across tumor regions. In the tumor core, reduced or dysfunctional pericyte coverage is associated with highly permeable, poorly perfused vessels and elevated interstitial pressure. In contrast, the invasive margin often retains subsets of pericytes with partial barrier-supporting capacity, creating regions of relative vascular integrity that can limit drug penetration and facilitate tumor cell survival (Li et al., 2024b). Emerging single-cell and spatial transcriptomic studies further support the existence of distinct pericyte subpopulations with divergent effects on vascular permeability (Decker et al., 2025; Sakai et al., 2025).

Functionally, this pericyte-driven heterogeneity has important therapeutic implications. The persistence of pericyte-supported vascular niches may underlie resistance to anti-angiogenic therapies by maintaining perfusion despite endothelial targeting (Cheng et al., 2023; Lv et al., 2024; Martinez-Morga et al., 2024). At the same time, excessive pericyte dysfunction contributes to vascular leakiness and inefficient drug delivery (Braun et al., 2025). This dual role highlights a central challenge in GBM therapy: whether pericytes should be targeted, preserved, or functionally reprogrammed. Consequently, current strategies are increasingly focused on vascular normalization, aiming to restore balanced pericyte-endothelial interactions and reduce barrier heterogeneity (Iesato and Nucera, 2021; Sattiraju and Mintz, 2019). However, the extent to which such normalization can be achieved in the highly plastic GBM microenvironment remains uncertain.

#### 4.4.2 Breast cancer: pericytes in metastatic niche formation and vascular remodeling

Breast cancer, particularly triple-negative breast cancer (TNBC), exhibits a strong propensity for brain metastasis, a process that requires extensive remodeling of the cerebral microenvironment prior to tumor cell colonization. Increasing evidence suggests that pericytes are actively involved in the establishment of a permissive pre-metastatic niche, rather than being passive components of BBB (Geissler et al., 2023; Sattiraju and Mintz, 2019).

Tumor-derived systemic factors play role in this process. Secreted molecules such as lysyl oxidase (LOX) and VEGF can act distally to reprogram brain-resident pericytes, altering their phenotype and weakening their interactions with endothelial cells (Ribeiro et al., 2017). This remote conditioning disrupts BBB integrity and promotes localized vascular permeability, thereby facilitating the extravasation of circulating tumor cells (CTCs) into the brain parenchyma. Following metastatic seeding, pericytes continue to shape the vascular architecture within metastatic lesions. Aberrant pericyte-endothelial interactions give rise to a vascular phenotype that is neither fully normalized nor completely disrupted, but instead characterized by selective permeability (Lyle et al., 2016; Mu et al., 2024). Such “leaky yet restrictive” vessels may provide a protective niche for metastatic tumor cells by permitting nutrient exchange while limiting immune cell infiltration and drug penetration.

In addition to their structural roles, pericytes contribute to the immunological remodeling of the metastatic microenvironment. By secreting chemokines such as CXCL12, pericytes can recruit tumor-associated macrophages (TAMs), thereby promoting an immunosuppressive milieu that supports metastatic growth (Navarro et al., 2016). This dual role-modulating both vascular function and immune cell recruitment positions pericytes may represent important contributors to the establishment and maintenance of brain metastatic niches.

Collectively, these findings highlight that pericytes are not merely disrupted during metastasis but are actively co-opted to facilitate tumor cell colonization, vascular adaptation, and immune evasion (Ascierto et al., 2019; Moro et al., 2024). Targeting pericyte-mediated niche formation may in the context, therefore represent a promising, yet underexplored, therapeutic strategy for preventing or treating brain metastases in TNBC.

#### 4.4.3 Pancreatic cancer: pericyte-mediated desmoplasia and hypovascular barrier

Pancreatic ductal adenocarcinoma (PDAC) is characterized by a dense desmoplastic stroma and profound hypovascularity, representing a distinct form of tumor-associated barrier dysfunction that contrasts sharply with the hyperpermeable vasculature observed in other malignancies (Feig et al., 2012; Hosein et al., 2022). In this context, pericytes contribute not only to vascular regulation but also to stromal remodeling, thereby playing a dual role in shaping the tumor microenvironment.

Emerging evidence suggests that tumor-associated pericytes in PDAC can undergo phenotypic transition toward fibroblast-like states, contributing to the pool of cancer-associated fibroblasts (CAFs) (Hosaka et al., 2016; LeBleu and Kalluri, 2018; Wang et al., 2021). This pericyte-to-fibroblast transition amplifies ECM deposition, including collagen and hyaluronan, leading to excessive matrix stiffness and increased interstitial fluid pressure (DuFort et al., 2016). As a consequence, the already sparse tumor vasculature becomes further compressed, often associated with reduced perfusion and impaired nutrient and drug delivery (Nieskoski et al., 2017).

Unlike the leaky vascular barriers observed in the brain such as BBB or BRB under pathological conditions PDAC exhibits a functionally restrictive barrier defined by hypoperfusion rather than hyperpermeability (Ligorio et al., 2019; Olive et al., 2009). In this setting, pericytes exert paradoxical effects: while their detachment or dysfunction can destabilize microvessels, their transdifferentiation into matrix-producing CAFs exacerbates stromal stiffening and vascular collapse. Thus, pericytes contribute both indirectly and directly to the formation of a physical barrier that limits therapeutic penetration (Vennin et al., 2018).

This context-dependent role highlights a fundamental principle of pericyte biology: barrier dysfunction does not uniformly equate to increased permeability. Instead, pericyte dysregulation can contribute to divergent vascular phenotypes, ranging from excessive leakiness to extreme restriction, depending on the tissue and tumor context (Carmeliet and Jain, 2011; Jain, 2014). In PDAC, targeting stromal remodeling and pericyte-derived CAF populations has therefore emerged as a potential strategy to decompress vessels, restore perfusion, and improve drug delivery although clinical translation remains challenging.

## 5 Key unanswered questions and future directions

Despite significant advances in pericyte biology, several fundamental questions remain unresolved and continue to define the field’s future direction. A major challenge is the lack of truly specific and universally applicable markers for pericyte identification across tissues and vascular beds (Simmonds et al., 2024). Although combinatorial marker strategies have improved resolution compared with single-marker approaches, they remain insufficient to clearly distinguish pericytes from vascular smooth muscle cells, fibroblasts, and other perivascular populations (Yao, 2022). Developing more refined genetic tools, such as intersectional lineage-tracing systems and subtype-specific reporter lines, will be essential for improving cellular specificity and experimental precision. Another important unresolved issue is whether pericyte heterogeneity reflects stable lineage-defined subpopulations or dynamic phenotypic states driven by local microenvironmental cues. Addressing this will require longitudinal lineage-tracing combined with single-cell multi-omics and spatial transcriptomics across disease progression (Bi et al., 2025). Closely related is the need to define the molecular and epigenetic mechanisms govering whether pericytes adopt protective or pathogenic roles. Identifying the regulatory programs underlying these functional switches may uncover context-dependent therapeutic targets. A further challenge is to elucidate how pericytes integrate signals from endothelial cells, astrocytes, immune cells, and extracellular matrix (Paszek et al., 2005). Moving from simplified pairwise interactions toward systems-level analyses will be essential to capture the emergent vascular signaling networks. From a translational perspective, it remains unclear whether pericyte-targeted therapies can achieve sufficient specificity and safety in humans, given the widespread distribution and functional diversity (Jaime Garcia et al., 2025). Improved delivery systems, physiologically relevant disease models, and systematic evaluation of potential off-target effects are needed. In addition, the generalizability of CNS pericytes findings to other organs remains unresolved (Nakanishi et al., 2024). Comparative studies across vascular beds using standardized methodologies will be important to define conserved *versus* tissue-specific features.

Finally, the impact of aging on pericyte heterogeneity and function remains insufficiently understood and may represent an important determinant of vascular disease susceptibility and cancer progression. Addressing these questions will require integrated multidisciplinary approaches combining genetic models, multi-omics technologies, vascular organoid and organ-on-chip systems, *in vivo* imaging, and computational modeling.

## 6 Conclusions and future perspectives

Pericytes should be viewed not as isolated master regulators but as integral components of multicellular network in which endothelial cells, astrocytes, immune cells, and extracellular matrix collectively determine vascular behavior. In this review, we have highlighted pericytes as important signaling nodes within microenvironmental signaling crosstalk that govern vascular barrier states across physiological and pathological contexts. Rather than functioning as passive structural components, pericytes dynamically involved in the integration of biochemical, mechanical, inflammatory, and metabolic cues through multidimensional interactions with endothelial cells, immune cells, and the extracellular matrix (Jordan et al., 2026). This integrative capacity positions pericytes as important regulators of vascular homeostasis and as participate in the development of disease-specific vascular phenotypes. Importantly, a unifying concept emerging from current evidence is that pericyte dysfunction does not lead to a single, uniform outcome. Instead, it contributes to context-dependent vascular responses ranging from hyperpermeability and barrier disruption to hypoperfusion and structural restriction (Zhao et al., 2024). This variability reflects both the complexity of the microenvironmental signals that pericytes integrate and their intrinsic heterogeneity across tissues and disease states (Huang, 2020). Consequently, understanding pericytes as components of a dynamic signaling network rather than isolated effectors provides a more comprehensive framework for interpreting vascular dysfunction.

Despite significant advances, several key challenges remain. While single-cell RNA sequencing (scRNA-seq) and spatial transcriptomic technologies have greatly expanded our understanding of vascular cell heterogeneity, several limitations should be considered when interpreting these datasets (Li et al., 2024c). Cell identity annotation remains inherently challenging, as pericyte populations often occupy transcriptional states closely overlapping with vascular smooth muscle cells and fibroblasts, and their classification is highly sensitive to the choice of marker genes and analytical pipelines (Guarino et al., 2025). In addition, transcript abundance does not necessarily reflect protein expression or functional activity, owing to layers of post-transcriptional regulation, translational control, and differences in protein stability and turnover. This decoupling becomes particularly relevant in highly plastic cell types such as pericytes, where transcriptional states may not directly correspond to functional phenotypes shaped by dynamic microenvironmental cues. Moreover, dissociation-based single-cell approaches may introduce sampling bias by preferentially capturing certain pericyte subpopulations while losing others that are more fragile or tightly embedded within the vascular niche (Kearns et al., 2023; Vanlandewijck et al., 2018a). Taken together, these limitations highlight the need for cautious interpretation of transcriptomic data and underscore the importance of integrating scRNA-seq and spatial profiling with proteomic, epigenomic, and functional validation strategies to more accurately infer biological mechanisms. In parallel, biomimetic systems, such as organ-on-a-chip platforms and 3D vascularized models, may provide more physiologically relevant tools to study pericyte function under controlled microenvironmental conditions. Furthermore, computational modeling and systems biology approaches hold promise for deciphering the complex signal integration processes that underlie pericyte behavior (Hanada et al., 2025). From a translational perspective, these insights call for a shift from single-pathway targeting toward context-specific modulation of pericyte function. Despite increasing interest in targeting pericyte-mediated signaling, clinical translation remains limited by the lack of pericyte-specific tools, pronounced cellular heterogeneity, and overlapping molecular features with other perivascular cells. Pericyte functions are also highly context-dependent, encompassing both vascular stabilization and contributions to inflammation, fibrosis, and pathological remodeling, raising the risk of context-specific and potentially divergent therapeutic outcomes. In addition, the long-term consequences of sustained pericyte modulation remain unclear. Collectively, these challenges underscore the need for precise, context-aware strategies and more physiologically relevant models before clinical application. Therapeutic strategies should aim not merely to enhance or inhibit pericyte activity, but to restore balanced microenvironmental signaling and vascular homeostasis. In this regard, combining vascular-targeted therapies with approaches that modulate immune responses, metabolic states, or extracellular matrix dynamics may offer improved efficacy across diverse diseases (He et al., 2025).

In conclusion, pericytes represent important signaling nodes within the broader vascular regulatory network, bridging multiple signaling domains and contributing to context-dependent vascular responses alongside endothelial cells, immune cells, and other components of the microenvironment. Continued efforts to unravel their integrative mechanisms and functional diversity will not only deepen our understanding of vascular biology but also open new avenues for precision therapies targeting complex microenvironmental networks in vascular diseases and cancer.
